# Supplementary material for: The cell-adhesion molecule Echinoid promotes tissue survival and separately restricts tissue overgrowth
Source: Development. 2025 Aug 7;152(15):dev204572. doi: 10.1242/dev.204572 (PMC12377819; doi:10.1242/dev.204572)
Supplement: Supplementary information [file develop-152-204572-s1.pdf]

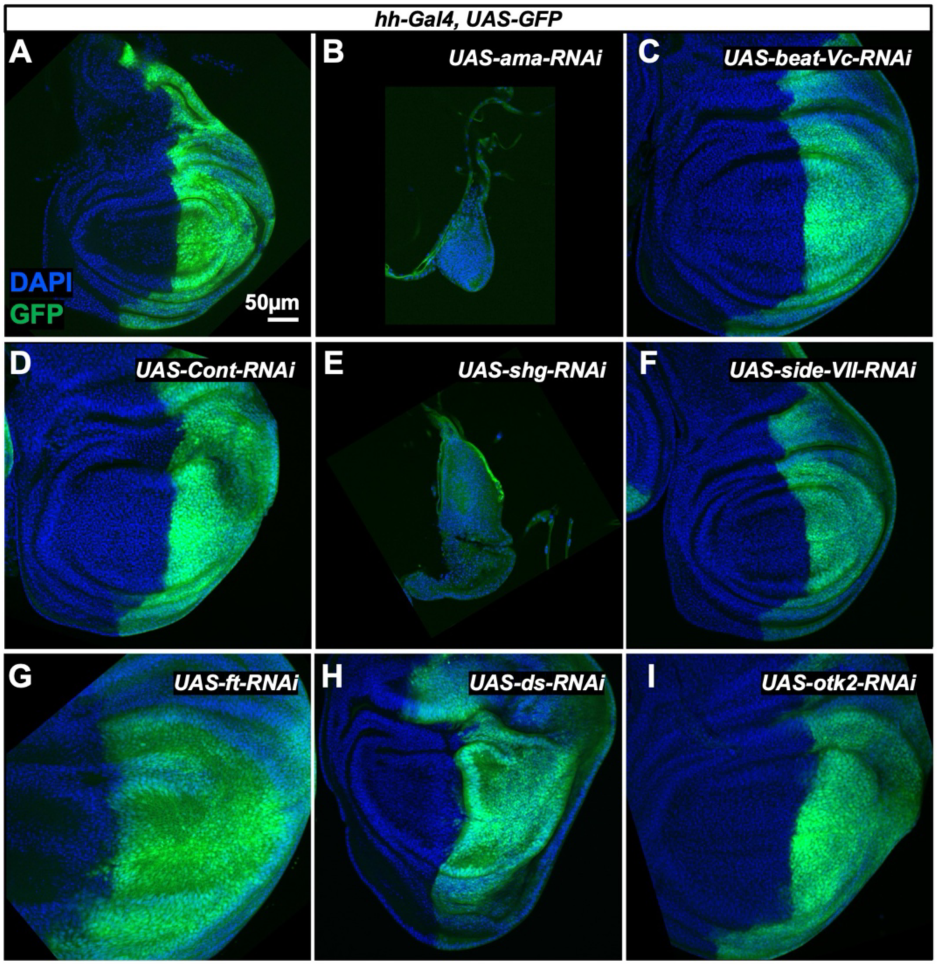

Fig. S1. Effects of gene knockdowns in the posterior compartment.

(**A-I**) Phenotype of wing imaginal discs with posterior compartment knockdown of genes identified as hits in the clonal screen. *hh-Gal4* drives expression of *UAS-GFP* alone (**A**) or *UAS-GFP* and RNAi transgenes (**B-I**). Knockdown of *ama* (**B**) or *shg* (**E**) resulted in absent or severely undergrown discs with little or no identifiable posterior tissue. Knockdown of *beat-Vc* (**C**), *Cont* (**D**), *side-VII* (**F**), *ft* (**G**), *ds* (**H**), and *otk2* (**I**) did not prevent the survival of cells in the posterior compartment.

Development • Supplementary information

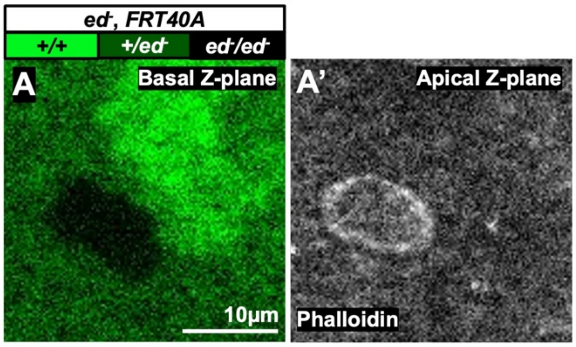

Fig. S2. An apical actomyosin cable is observed at the periphery of *ed* mutant clones.
(**A, A'**) An apical actomyosin cable is observed at the interface of *ed* mutant tissue and the wild type neighbors (*ed*^{lF20}/ *ed*^{lF20} mitotic recombination clone is shown), consistent with previous reports (Wei *et al.* 2005; Laplante and Nilson 2006, 2011; Lin *et al.* 2007; Chang *et al.* 2011). The panels show different Z-planes of the same image because the apical actomyosin cable is located at a Z-plane with weak GFP signal.

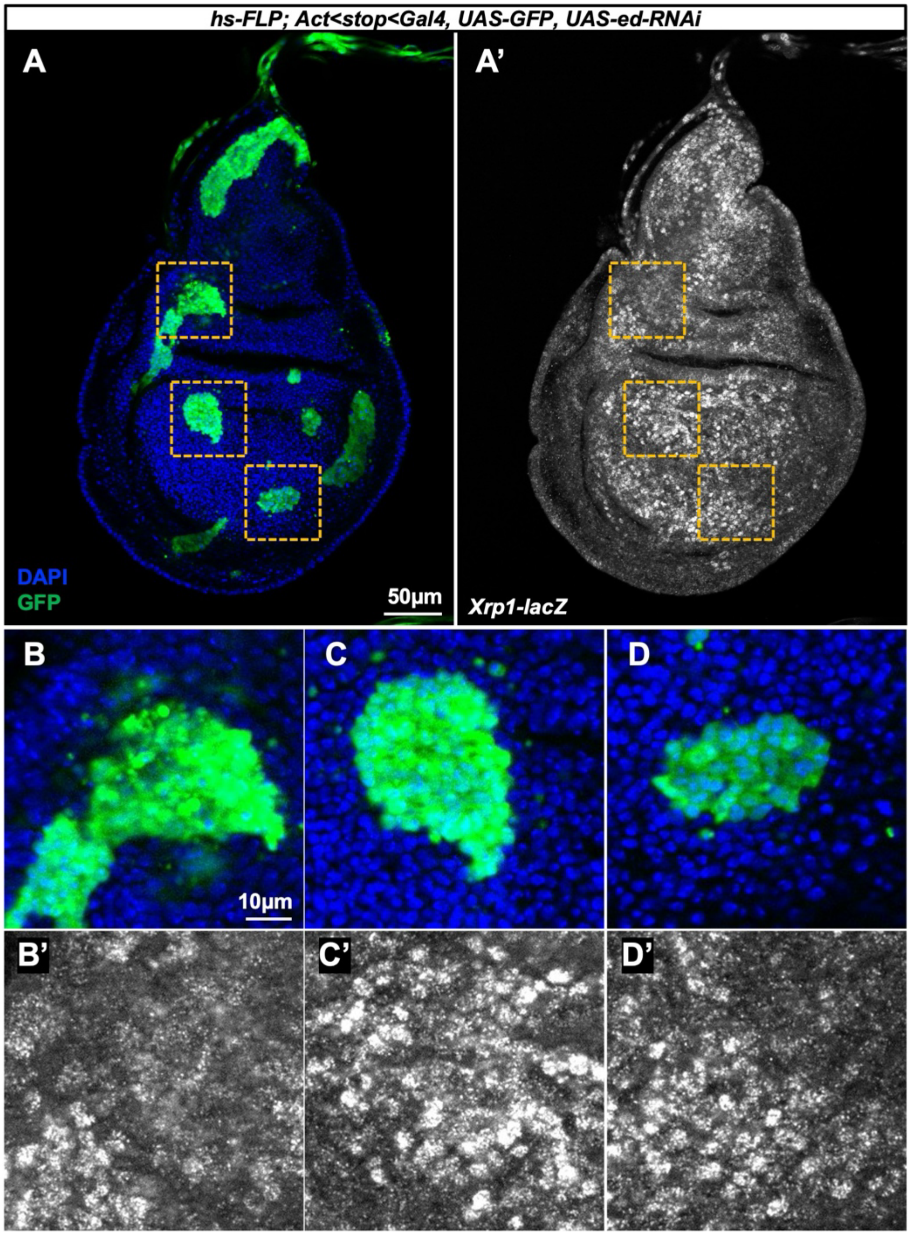

Fig. S3. Expression of *Xrp1-lacZ* is not altered in *ed-RNAi* clones.

(**A-D'**) Wing discs harboring an *Xrp1-lacZ* reporter containing clones of GFP-marked cells expressing ed-RNAi. Images of the entire disc are shown in (**A, A'**). The regions indicated by dashed lines are shown at higher magnification in (**B-D'**). Timing of clone generation and larval dissection were not as tightly controlled as in experiments intended to assess clone survival or morphology.

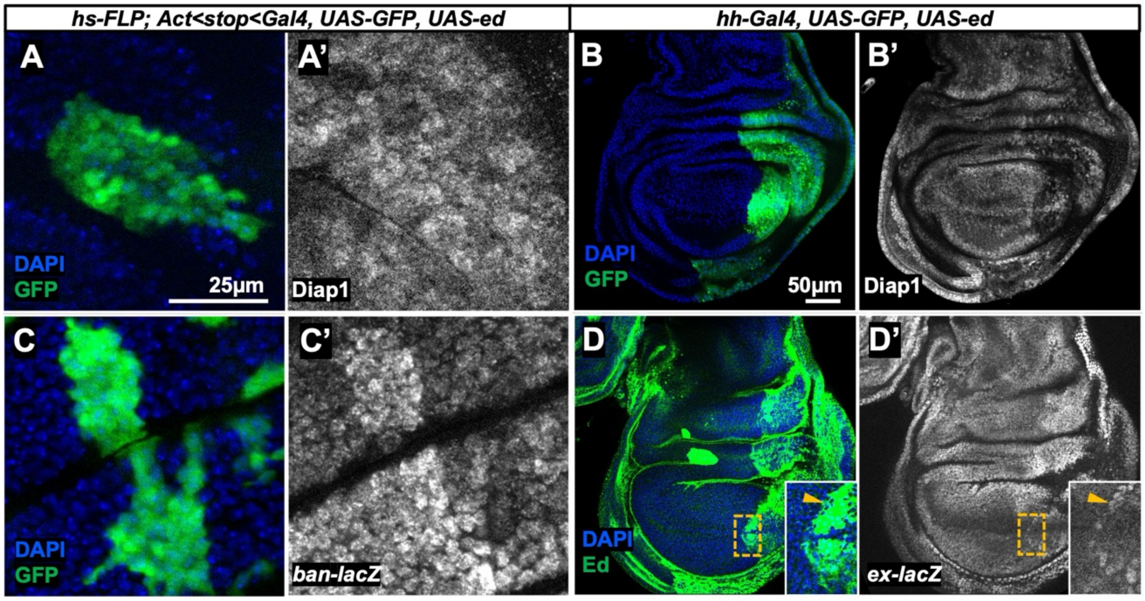

Fig. S4. Phenotypes resulting from *echinoid* overexpression.

(**A, B**) Effect of *ed* overexpression on Diap1 levels. GFP-marked clone that expresses *UAS-ed* (**A**) showing no obvious effect on Diap1 levels (**A'**). Overexpression of *ed* in the entire posterior compartment (**B**) results in a decrease in Diap1 (**B'**).

(**C**) Overexpression of *ed* in GFP-marked clones (**C**) results in an increase in *ban-lacZ* expression (**C'**).

(**D**) Overexpression of *ed* in the posterior compartment results in increased *ex-lacZ* expression (**D'**). The autonomous increase is less obvious in the pouch, except in the *ed*-overexpressing cells at the border with wild-type cells (inset, arrowhead).

Development • Supplementary information

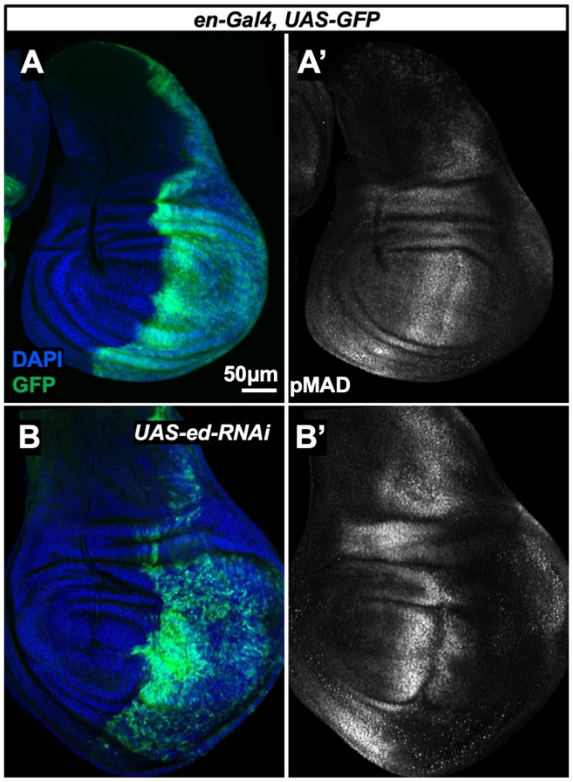

Fig. S5. Expression pattern of *pMAD* in discs with compartment-wide *echinoid* loss.
(**A-B**) *en-Gal4* drives expression of *UAS-GFP* (**A, A'**) or *UAS-GFP* and *UAS-ed-RNAi* (**B, B'**). Discs are stained with anti-pMAD (**A', B'**). All images are shown at the same scale (scale bar in panel **A**).

Development • Supplementary information

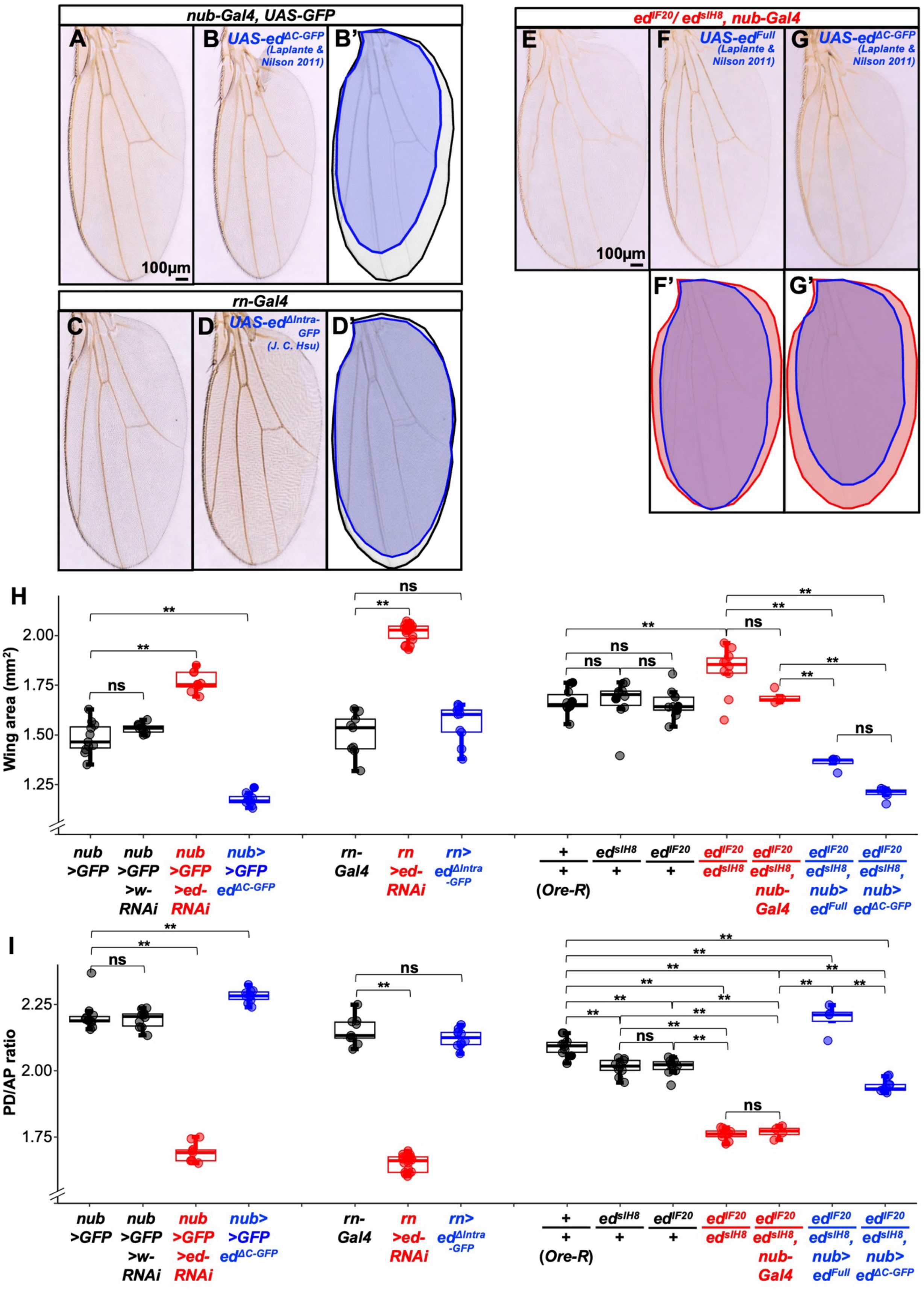

Development • Supplementary information

Fig. S6. Domain-specific effects of *echinoid* on adult wing size and shape.

(**A-D**) Effect of expressing *ed* transgenes where the cytoplasmic domain has been replaced by GFP (*ed*$^{\Delta C\text{-}GFP}$ *and ed*$^{\Delta Intra\text{-}GFP}$) on wing shape and size. Panels show adult wings of the indicated genotype. An overlay of (**B**) over (**A**) is shown in (B'). An overlay of (**D**) over (**C**) is shown in (**D'**).

(**E-I**) Effect of expressing *ed*^{Full} *ed*$^{\Delta C\text{-}GFP}$ on wing size and shape in otherwise *ed*-depleted wings. (**E**) Wings of the heteroallelic combination *ed*^{slH8}/*ed*^{lF20} that generate viable adults together with *nub-Gal4*. Inclusion of *UAS-ed*^{Full} (**F**) and *UAS-ed*$^{\Delta C\text{-}GFP}$ (**G**) reduces wing size and brings the aspect ratio closer to wild-type. (**F'**) and (**G'**) show overlays of (**F**) and (**G**) over (**E**), respectively.

(**H-I**) Quantification of wing areas (**H**) and PD/AP ratio (**I**). The same wings were used in (**H**) and (**I**); number of wings differ if damage or mounting artifacts prevented measurement of both wing area and PD/AP ratio ratio: *nub-Gal4, UAS-GFP* (n=11); *nub-Gal4, UAS-GFP, UAS-w-RNAi* (n=10); *nub-Gal4, UAS-GFP, UAS-ed-RNA* (n=10); *nub-Gal4, UAS-GFP, UAS-ed*$^{\Delta C\text{-}GFP}$ (n=10); *rn-Gal4* (n=9); *rn-Gal4, UAS-ed-RNAi* (n=19); *rn-Gal4, UAS-ed*$^{\Delta Intra\text{-}GFP}$ (n=10); *+/+* [*Oregon-R*] (n=10); *ed*^{slH8}/+ (n=10); *ed*^{lF20}/+ (n=10); *ed*^{slH8}/*ed*^{lF20} (n=10 in H; n=9 in I); *ed*^{slH8}/*ed*^{lF20}, *nub-Gal4* (n=4); *ed*^{slH8}/*ed*^{lF20}, *nub-Gal4, UAS-ed*^{Full} (n=4); *ed*^{slH8}/*ed*^{lF20}, *nub-Gal4, UAS-ed*$^{\Delta C\text{-}GFP}$ (n=6 in H; n=9 in I) Note data from the following genotypes are duplicated in (**H**) from Figure 7I: *nub-Gal4, UAS-GFP, UAS-w-RNAi; nub-Gal4, UAS-GFP, UAS-ed-RNAi; rn-Gal4; rn-Gal4, UAS-ed-RNAi; +/+* [*Oregon-R*]; *ed*^{slH8}/+; *ed*^{lF20}/+; *ed*^{slH8}/*ed*^{lF20}. "ns" indicates p> 0.05, ** indicates p< 0.01, calculated using ANOVA with post-hoc Tukey's HSD test.

Development • Supplementary information

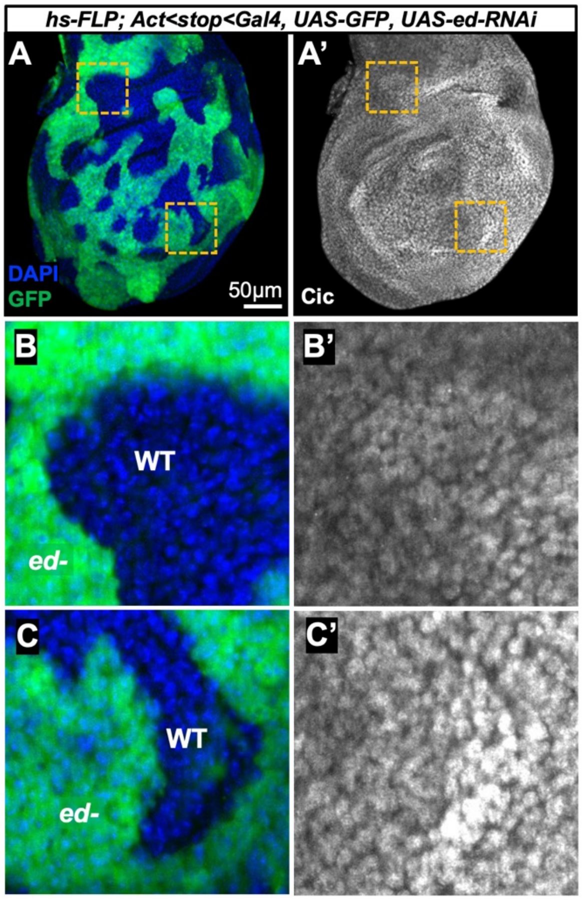

Fig. S7. Reduced Capicua levels in *ed* clones.

(**A-C**) Effect of *ed* knockdown on Capicua (Cic) levels. (**A**) Cic levels are lower in some *ed* clones than in wild-type neighbors, indicating increased EGFR activity within the clones. (**B, C**) and (**B', C'**) show GFP-marked clones and Cic antibody staining at higher magnification. The location of these clones is indicated in (**A, A'**) by dashed lines.

Table S1. Initial candidate list.

Gene abbreviation	Gene name	Type	Source
ama	amalgam	IgSF	Hynes 2000, Vogel 2003
babos	babos	IgSF	Hynes 2000, Vogel 2003
bdl	borderless	IgSF	Hynes 2000, Vogel 2003
beat-Ia	beaten path Ia	IgSF	Hynes 2000, Vogel 2003
beat-Ib	beaten path Ib	IgSF	Hynes 2000, Vogel 2003
beat-Ic	beaten path Ic	IgSF	Hynes 2000, Vogel 2003
beat-IIa	beaten path IIa	IgSF	Hynes 2000, Vogel 2003
beat-IIb	beaten path IIb	IgSF	Hynes 2000, Vogel 2003
beat-IIIa	beaten path IIIa	IgSF	Vogel 2003
beat-IIIb	beaten path IIIb	IgSF	Vogel 2003
beat-IIIc	beaten path IIIc	IgSF	Vogel 2003
beat-IV	beaten path IV	IgSF	Vogel 2003
beat-Va	beaten path Va	IgSF	Vogel 2003
beat-Vb	beaten path Vb	IgSF	Vogel 2003
beat-Vc	beaten path Vc	IgSF	Vogel 2003
beat-VI	beaten path VI	IgSF	Vogel 2003
beat-VII	beaten path VII	IgSF	Vogel 2003
boi	brother of ihog	IgSF	Hynes 2000, Vogel 2003
bsg	basigin	IgSF	Vogel 2003
bt	bent	IgSF	Hynes 2000, Vogel 2003
btl	breathless	IgSF	Hynes 2000, Vogel 2003
Cad74A	Cadherin 74A	Cadherin	Hynes 2000
Cad86C	Cadherin 86C	Cadherin	Hynes 2000
Cad87A	Cadherin 87A	Cadherin	Hynes 2000
Cad88C	Cadherin 88C	Cadherin	Hynes 2000
Cad89D	Cadherin 89D	Cadherin	Hynes 2000
Cad96Ca	Cadherin 96Ca	Cadherin	Hynes 2000
Cad96Cb	Cadherin 96Cb	Cadherin	Hynes 2000
Cad99C	Cadherin 99C	Cadherin	Hynes 2000
CadN	Cadherin-N	Cadherin	Hynes 2000
CadN2	Cadherin-N2	Cadherin	Hynes 2000
Cals	Calsyntenin-1	Cadherin	Hynes 2000

CG13506	CG13506	IgSF	Hynes 2000, Vogel 2003
CG13532	CG13532	IgSF	Hynes 2000, Vogel 2003
CG13992	CG13992	IgSF	Vogel 2003
CG15312	CG15312	IgSF	Hynes 2000, Vogel 2003
CG16974	CG16974	IgSF	Hynes 2000, Vogel 2003
CG17839	CG17839	IgSF	Hynes 2000, Vogel 2003
CG31431	CG31431	IgSF	Vogel 2003
CG33543	CG33543	IgSF	Hynes 2000, Vogel 2003
CG34353	CG34353	IgSF	Vogel 2003
CG44153	CG44153	IgSF	Vogel 2003
CG45263	CG45263	IgSF	Hynes 2000, Vogel 2003
CG5597	CG5597	IgSF	Hynes 2000, Vogel 2003
CG6867	CG6867	IgSF	Hynes 2000, Vogel 2003
CG7166	CG7166	IgSF	Hynes 2000, Vogel 2003
CG7607	CG7607	IgSF	Hynes 2000, Vogel 2003
Cont	Contactin	IgSF	Hynes 2000, Vogel 2003
DIP-alpha	Dpr-interacting protein alpha	IgSF	Vogel 2003
DIP-beta	Dpr-interacting protein beta	IgSF	Hynes 2000, Vogel 2003
DIP-delta	Dpr-interacting protein delta	IgSF	Hynes 2000, Vogel 2003
DIP-epsilon	Dpr-interacting protein epsilon	IgSF	Vogel 2003
DIP-eta	Dpr-interacting protein eta	IgSF	Hynes 2000, Vogel 2003
DIP-gamma	Dpr-interacting protein gamma	IgSF	Hynes 2000, Vogel 2003
DIP-iota	Dpr-interacting protein iota	IgSF	Hynes 2000, Vogel 2003
DIP-kappa	Dpr-interacting protein kappa	IgSF	Vogel 2003
DIP-lambda	Dpr-interacting protein lambda	IgSF	Manual curation
DIP-theta	Dpr-interacting protein theta	IgSF	Hynes 2000, Vogel 2003
DIP-zeta	Dpr-interacting protein zeta	IgSF	Hynes 2000, Vogel 2003
dpr1	defective proboscis extension response 01	IgSF	Vogel 2003
dpr10	defective proboscis extension response 10	IgSF	Hynes 2000, Vogel 2003
dpr11	defective proboscis extension response 11	IgSF	Hynes 2000, Vogel 2003
dpr12	defective proboscis extension response 12	IgSF	Hynes 2000, Vogel 2003
dpr13	defective proboscis extension response 13	IgSF	Hynes 2000, Vogel 2003

Development • Supplementary information

dpr14	defective proboscis extension response 14	IgSF	Hynes 2000, Vogel 2003
dpr15	defective proboscis extension response 15	IgSF	Hynes 2000, Vogel 2003
dpr16	defective proboscis extension response 16	IgSF	Hynes 2000, Vogel 2003
dpr17	defective proboscis extension response 17	IgSF	Hynes 2000, Vogel 2003
dpr18	defective proboscis extension response 18	IgSF	Hynes 2000, Vogel 2003
dpr19	defective proboscis extension response 19	IgSF	Vogel 2003
dpr2	defective proboscis extension response 02	IgSF	Hynes 2000, Vogel 2003
dpr20	defective proboscis extension response 20	IgSF	Hynes 2000, Vogel 2003
dpr21	defective proboscis extension response 21	IgSF	Manual curation
dpr3	defective proboscis extension response 03	IgSF	Hynes 2000, Vogel 2003
dpr4	defective proboscis extension response 04	IgSF	Hynes 2000, Vogel 2003
dpr5	defective proboscis extension response 05	IgSF	Hynes 2000, Vogel 2003
dpr6	defective proboscis extension response 06	IgSF	Manual curation
dpr7	defective proboscis extension response 07	IgSF	Vogel 2003
dpr8	defective proboscis extension response 08	IgSF	Hynes 2000, Vogel 2003
dpr9	defective proboscis extension response 09	IgSF	Hynes 2000, Vogel 2003
ds	dachsous	Cadherin	Hynes 2000
Dscam1	Down syndrome cell adhesion molecule 1	IgSF	Hynes 2000, Vogel 2003
Dscam2	Down syndrome cell adhesion molecule 2	IgSF	Hynes 2000, Vogel 2003
Dscam3	Down syndrome cell adhesion molecule 3	IgSF	Hynes 2000, Vogel 2003
Dscam4	Down syndrome cell adhesion molecule 4	IgSF	Hynes 2000, Vogel 2003
ed	echinoid	IgSF	Hynes 2000, Vogel 2003
Eph	Eph receptor tyrosine kinase	IgSF	Hynes 2000, Vogel 2003
Fas2	Fasciclin 2	IgSF	Hynes 2000, Vogel 2003
Fas3	Fasciclin 3	IgSF	Vogel 2003
fipi	factor of interpulse interval	IgSF	Hynes 2000, Vogel 2003
fra	frazzled	IgSF	Hynes 2000, Vogel 2003

Development • Supplementary information

fred	friend of echinoid	IgSF	Hynes 2000, Vogel 2003
ft	fat	Cadherin	Hynes 2003
hbs	hibris	IgSF	Hynes 2000, Vogel 2003
hig	hikaru genki	IgSF	Hynes 2000, Vogel 2003
htl	heartless	IgSF	Hynes 2000, Vogel 2003
ihog	interference hedgehog	IgSF	Hynes 2000, Vogel 2003
Imp-L2	Ecdysone-inducible gene L2	IgSF	Hynes 2000, Vogel 2003
kek1	kekkon-1	IgSF	Hynes 2000, Vogel 2003
kek2	kekkon-2	IgSF	Hynes 2000, Vogel 2003
kek3	kekkon-3	IgSF	Hynes 2000, Vogel 2003
kek4	kekkon4	IgSF	Hynes 2000, Vogel 2003
kek5	kekkon5	IgSF	Hynes 2000, Vogel 2003
kek6	kekkon 6	IgSF	Vogel 2003
kirre	kin of irre	IgSF	Hynes 2000, Vogel 2003
klg	klingon	IgSF	Hynes 2000, Vogel 2003
Kug	kugelei	Cadherin	Hynes 2000
Lac	Lachesin	IgSF	Hynes 2000, Vogel 2003
Lar	Leukocyte-antigen-related-like	IgSF	Vogel 2003
lbk	lambik	IgSF	Vogel 2003
MnM	myomesin and myosin binding protein	IgSF	Hynes 2000, Vogel 2003
nepl6	Neprilysin-like 6	IgSF	Hynes 2000
nkt	noktochor	IgSF	Hynes 2000, Vogel 2003
nolo	no long nerve cord	IgSF	Hynes 2000, Vogel 2005
Nrg	Neuroglian	IgSF	Hynes 2000, Vogel 2003
nrm	neuromusculin	IgSF	Hynes 2000, Vogel 2003
otk	off-track	IgSF	Hynes 2000, Vogel 2003
otk2	off-track2	IgSF	Hynes 2000, Vogel 2003
plum	plum	IgSF	Hynes 2000, Vogel 2003
ppk12	pickpocket 12	IgSF	Vogel 2003
ppn	papilin	IgSF	Vogel 2003
Ptp69D	Protein tyrosine phosphatase 69D	IgSF	Hynes 2000, Vogel 2003
Pvr	PDGF- and VEGF-receptor related	IgSF	Hynes 2000, Vogel 2003
Pxn	Peroxidasin	IgSF	Hynes 2000, Vogel 2003
Remo	Remoulade	IgSF	Vogel 2003

Ret	Ret oncogene	Cadherin	Manual curation
robo1	roundabout 1	IgSF	Hynes 2000, Vogel 2003
robo2	roundabout 2	IgSF	Hynes 2000, Vogel 2003
robo3	roundabout 3	IgSF	Hynes 2000, Vogel 2003
rst	roughest	IgSF	Hynes 2000, Vogel 2003
sdk	sidekick	IgSF	Hynes 2000, Vogel 2003
Sema2a	Semaphorin 2a	IgSF	Vogel 2003
shg	shotgun	Cadherin	Hynes 2000
side	sidestep	IgSF	Hynes 2000, Vogel 2003
side-II	sidestep II	IgSF	Hynes 2000, Vogel 2003
side-III	sidestep III	IgSF	Hynes 2000, Vogel 2003
side-IV	sidestep IV	IgSF	Hynes 2000, Vogel 2003
side-V	sidestep V	IgSF	Hynes 2000, Vogel 2003
side-VI	sidestep VI	IgSF	Vogel 2003
side-VII	sidestep VII	IgSF	Hynes 2000, Vogel 2003
side-VIII	sidestep VIII	IgSF	Hynes 2000, Vogel 2003
sls	sallismus	IgSF	Hynes 2000, Vogel 2003
sns	sticks and stones	IgSF	Hynes 2000, Vogel 2003
stan	starry night	Cadherin	Hynes 2000
Strn-Mlck	Stretchin-Mlck	IgSF	Hynes 2000, Vogel 2003
tei	teiresias	IgSF	Hynes 2000, Vogel 2003
trol	terribly reduced optic lobes	IgSF	Vogel 2003
tutl	turtle	IgSF	Hynes 2000, Vogel 2003
unc-5	unc-5	IgSF	Hynes 2000, Vogel 2003
Unc-89	Unc-89	IgSF	Hynes 2000, Vogel 2003
vn	vein	IgSF	Hynes 2000, Vogel 2003
wrapper	wrapper	IgSF	Hynes 2000, Vogel 2003
zormin	zormin	IgSF	Hynes 2000, Vogel 2003

Available for download at
https://journals.biologists.com/dev/article-lookup/doi/10.1242/dev.204572#supplementary-data

Table S2. Genes excluded from screen.

Gene abbrev.	Gene name	Criteria for exclusion
ppk12	pickpocket 12	Known to not be adhesion molecule (ENaC subunit); Incorrectly classified as IgSF?
btl	breathless	Known to not be adhesion molecule (RTK)
htl	heartless	Known to not be adhesion molecule (RTK)
Ret	Ret oncogene	Known to not be adhesion molecule (RTK)
bt	bent	Known to not be adhesion molecule (sarcomere component)
sls	sallismus	Known to not be adhesion molecule (sarcomere component)
Strn-Mlck	Stretchin-Mlck	Known to not be adhesion molecule (sarcomere component)
Unc-89	Unc-89	Known to not be adhesion molecule (sarcomere component)
zormin	zormin	Known to not be adhesion molecule (sarcomere component)
Sema2a	Semaphorin 2a	Known to not be adhesion molecule (secreted)
beat-Ia	beaten path Ia	No expression RNAseq detected in wing disc
beat-Ib	beaten path Ib	No expression RNAseq detected in wing disc
beat-Ic	beaten path Ic	No expression RNAseq detected in wing disc
Cad88C	Cadherin 88C	No expression RNAseq detected in wing disc
Cad89D	Cadherin 89D	No expression RNAseq detected in wing disc
CadN	Cadherin-N	No expression RNAseq detected in wing disc
CadN2	Cadherin-N2	No expression RNAseq detected in wing disc
CG13532	CG13532	No expression RNAseq detected in wing disc
CG17839	CG17839	No expression RNAseq detected in wing disc
CG31431	CG31431	No expression RNAseq detected in wing disc
CG6867	CG6867	No expression RNAseq detected in wing disc
DIP-beta	Dpr-interacting protein beta	No expression RNAseq detected in wing disc
DIP-delta	Dpr-interacting protein delta	No expression RNAseq detected in wing disc
DIP-eta	Dpr-interacting protein eta	No expression RNAseq detected in wing disc
DIP-gamma	Dpr-interacting protein gamma	No expression RNAseq detected in wing disc
DIP-iota	Dpr-interacting protein iota	No expression RNAseq detected in wing disc
DIP-theta	Dpr-interacting protein theta	No expression RNAseq detected in wing disc
DIP-zeta	Dpr-interacting protein zeta	No expression RNAseq detected in wing disc
dpr10	defective proboscis extension response 10	No expression RNAseq detected in wing disc

dpr11	defective proboscis extension response 11	No expression RNAseq detected in wing disc
dpr12	defective proboscis extension response 12	No expression RNAseq detected in wing disc
dpr13	defective proboscis extension response 13	No expression RNAseq detected in wing disc
dpr15	defective proboscis extension response 15	No expression RNAseq detected in wing disc
dpr2	defective proboscis extension response 02	No expression RNAseq detected in wing disc
dpr20	defective proboscis extension response 20	No expression RNAseq detected in wing disc
dpr3	defective proboscis extension response 03	No expression RNAseq detected in wing disc
dpr4	defective proboscis extension response 04	No expression RNAseq detected in wing disc
dpr5	defective proboscis extension response 05	No expression RNAseq detected in wing disc
dpr8	defective proboscis extension response 08	No expression RNAseq detected in wing disc
Dscam2	Down syndrome cell adhesion molecule 2	No expression RNAseq detected in wing disc
Dscam3	Down syndrome cell adhesion molecule 3	No expression RNAseq detected in wing disc
Dscam4	Down syndrome cell adhesion molecule 4	No expression RNAseq detected in wing disc
fipi	factor of interpulse interval	No expression RNAseq detected in wing disc
kek3	kekkon-3	No expression RNAseq detected in wing disc
kek4	kekkon4	No expression RNAseq detected in wing disc
klg	klingon	No expression RNAseq detected in wing disc
nepl6	Neprilysin-like 6	No expression RNAseq detected in wing disc; Incorrectly classified as IgSF?
nolo	no long nerve cord	No expression RNAseq detected in wing disc
robo3	roundabout 3	No expression RNAseq detected in wing disc
side-II	sidestep II	No expression RNAseq detected in wing disc
side-III	sidestep III	No expression RNAseq detected in wing disc
side-VI	sidestep VI	No expression RNAseq detected in wing disc
side-VIII	sidestep VIII	No expression RNAseq detected in wing disc

Available for download at
https://journals.biologists.com/dev/article-lookup/doi/10.1242/dev.204572#supplementary-data

Table S3. Results from screen.

Gene abbrev.	Gene name	Lines	Result (-- = not a hit)	Notes
ama	amalgam	BL 33416	Undergrowth (severe)	Screened with TIE-DYE
babos	babos	BL 36728	--	
bdl	borderless	V 4806	-- see note	Some clones on the smaller side, but not convincing
beat-IIb	beaten path IIb	BL 57157	--	
beat-IIIa	beaten path IIIa	BL 64526	--	
beat-IIIb	beaten path IIIb	BL 56984<br>V 36237	-- see note<br>--	Initially thought there could be a slight difference of clone density A/P compartments, but was not replicable
beat-IIIc	beaten path IIIc	BL 50941	--	
beat-IV	beaten path IV	V 52413	--	
beat-Va	beaten path Va	BL 60053	--	
beat-Vb	beaten path Vb	V 17832	--	
beat-Vc	beaten path Vc	BL 60067	Undergrowth (severe)	Few clones survive
beat-VI	beaten path VI	V 6694	--	
beat-VII	beaten path VII	BL 60056	--	
boi	brother of ihog	V 29592	--	
bsg	basigin	BL 52110<br>V 2789	--<br>--	
Cad74A	Cadherin 74A	V 36320	--	
Cad86C	Cadherin 86C	BL 53314<br>BL 61280	--<br>--	
Cad96Ca	Cadherin 96Ca	BL 55877	--	Screened with TIE-DYE
Cad99C	Cadherin 99C	BL 35037	--	
CG13992	CG13992	V 2642	--	
CG15312	CG15312	V 101286	--	
CG16974	CG16974	BL 42590	--	
CG33543	CG33543	BL 64879<br>V 17859	--<br>--	
CG34353	CG34353	V 22788	--	
CG44153	CG44153	BL 33350	--	Screened with TIE-DYE
CG45263	CG45263	BL 62468<br>V 18706	--<br>--	
CG5597	CG5597	V 12875	--	

Development • Supplementary information

CG7166	CG7166	V 27116	--	
Cont	Contactin	BL 34867	Undergrowth (Severe)	
DIP-alpha	Dpr-interacting protein alpha	V 17116	--	
DIP-lambda	Dpr-interacting protein lambda	BL 41980	--	
dpr1	defective proboscis extension response 01	V 26879<br>V 27087	-- see note<br>--	3/6 discs had slightly low clone size/density
dpr14	defective proboscis extension response 14	V 8005	--	
dpr16	defective proboscis extension response 16	V 31986<br>V 102628	--<br>--	
dpr18	defective proboscis extension response 18	V 983	--	
dpr6	defective proboscis extension response 06	V 41161	--	
ds	dachsous	BL 32964	Clones in pouch not elongated, round clones	
Dscam1	Down syndrome cell adhesion molecule 1	BL 38945	--	
ed	echinoid	V 938<br>V 3087<br>V 104279	Undergrowth (slight), round, smooth<br>Undergrowth (moderate), round, smooth<br>Undergrowth (severe), round, smooth	
Fas2	Fasciclin 2	BL 34084	--	
Fas3	Fasciclin 3	BL 77396	--	Maybe slight undergrowth, but not convincing
fra	frazzled	BL 40826	--	
fred	friend of echinoid	BL 42621	--	
ft	fat	BL 34970	Round clones	
hig	hikaru genki	BL 42000	--	
ihog	interference hedgehog	BL 64541	--	
Imp-L2	Ecdysone-inducible gene L2	BL 64936	--	Screened with TIE-DYE
kek2	kekkon-2	V 4745	--	
kek5	kekkon5	BL 40830	--	
kirre	kin of irre	BL 64918	--	

Development • Supplementary information

Lar	Leukocyte-antigen-related-like	BL 40938	--	
lbk	lambik	BL 28903	--	
MnM	myomesin and myosin binding protein	BL 65245<br>V 43603	--<br>--	
nkt	noktochor	V 43018<br>V 43017	--<br>--	
Nrg	Neuroglian	BL 37496	--	
otk	off-track	BL 55869	--	
otk2	off-track2	BL 55892	Clones underrepresented especially in pouch; Rounded, cyst-like clones observed in hinge	Screened with TIE-DYE
plum	plum	BL 60062	--	
ppn	papilin	V 41901<br>V 16523	--<br>--	
Pvr	PDGF- and VEGF-receptor related	BL 37520<br>V 977	--<br>--	
Pxn	Peroxidasin	V 15276	--	
Remo	Remolaude	BL 42497	--	
robo2	roundabout 2	BL 34589	--	Screened with TIE-DYE
sdk	sidekick	BL 33412	--	Screened with TIE-DYE
shg	shotgun	BL 32904	Undergrowth (severe)	
side	sidestep	BL 50642<br>V 1283	--<br>--	Screened with TIE-DYE
side-IV	sidestep IV	V 29806<br>V 16636	--<br>--	
side-V	sidestep V	V 44997	--	
side-VII	sidestep VII	V 10011	Undergrowth (small clones)	
sns	sticks and stones	BL 64872<br>V 877	-- see note<br>--	Clones maybe slightly smaller and rounder, but not convincing
stan	starry night	BL 35050	--	
tei	teiresias	V 42236	--	
unc-5	unc-5	V 8137	--	
wrapper	wrapper	BL 29561	--	Screened with TIE-DYE

Development • Supplementary information
